# Diet composition and environmental niche drive parasitic Syndiniales interactions with crustacean zooplankton

**DOI:** 10.1093/ismeco/ycaf248

**Published:** 2025-12-24

**Authors:** Neea Hanström, Kinlan M G Jan, Baptiste Serandour, Tianshuo Xu, Monika Winder

**Affiliations:** Department of Ecology, Environment, and Plant Sciences, Stockholm University, Stockholm, Sweden; Department of Ecology, Environment, and Plant Sciences, Stockholm University, Stockholm, Sweden; Department of Ecology, Environment, and Plant Sciences, Stockholm University, Stockholm, Sweden; Department of Ecology, Environment, and Plant Sciences, Stockholm University, Stockholm, Sweden; Department of Ecology, Environment, and Plant Sciences, Stockholm University, Stockholm, Sweden

**Keywords:** Syndiniales, parasitism, zooplankton, DNA metabarcoding, diet composition, environmental niche

## Abstract

Syndiniales are a group of parasitic dinoflagellates belonging to marine alveolates that infect a wide range of planktonic taxa, including other protists and metazoans. Sequence-based correlation networks have revealed that although Syndiniales account for the majority of links associated with mesozooplankton. However, these studies have not determined whether these parasite–zooplankton interactions result from overlapping environmental niches, direct infection, or indirect transmission via intake of infected prey items. To address these open questions, we identified associations between Syndiniales and zooplankton from selected copepod and cladoceran taxa using DNA metabarcoding. We further identified the role of prey consumption and environmental factors shaping Syndiniales–zooplankton associations across a brackish-to-marine environmental gradient. Our results show that Syndiniales are highly associated with zooplankton, but their association varies and is shaped by diet and environmental conditions. Syndiniales groups I and II were associated with all zooplankton species studied. The transmission to zooplankton likely occurs through secondary intake of infected prey, although direct ingestion of dinospores cannot be excluded. In contrast, Syndiniales group IV showed more specific associations, with copepods being the most likely final hosts given the high read abundance detected in copepods and previously described parasitic associations with crustaceans. Low oxygen concentration enhanced associations between Syndiniales group IV and copepods, whereas groups I and II showed the opposite pattern, indicating that environmental variables create distinct ecological niches for both the hosts and the parasites, driving niche-specific associations. These findings suggest that parasitic Syndiniales play a key role in planktonic food webs by redirecting carbon cycling.

## Introduction

Parasitism is a prevalent symbiotic form of life in most ecosystems, including marine environments [1–4]. Parasite infections affect the host’s fitness by lowering the lifespan and fecundity and increasing mortality [2, 4]. Parasite activity can reduce energy and nutrient transfer to upper trophic levels by releasing organic material from hosts and fueling the microbial loop [2, 4, 5]. Therefore, parasites play important roles in carbon cycling. Yet, parasitism is seldom accounted for as a mortality source in plankton food web models. Parasite dynamics and host-specificity are often overlooked due to difficulty in detecting and identifying parasites with visual inspection, as they are usually hidden within their hosts. DNA sequencing of the eukaryotic plankton community has advanced the understanding of protist–parasite occurrence and has become a powerful tool to reveal this diversity [6]. DNA metabarcoding studies have uncovered a high abundance and diversity of eukaryotic parasites belonging to Syndiniales (marine alveolates) at the global scale that frequently form up to half of 18S rRNA sequences and often dominate interactions with diverse plankton taxa [6–8]. Sequence-based correlation networks reveal that Syndiniales account for the majority of links associated with mesozooplankton organisms, mainly dominated by copepod species that form an important link between primary producers and upper trophic levels [7]. While these networks demonstrate the importance of Syndiniales and identify potential parasitoid top-down effects on zooplankton, they do not differentiate whether the parasite–zooplankton interactions result from overlapping environmental niches between the taxa or from direct interactions through prey consumption.

Syndiniales, early-branching dinoflagellates that belong to the eukaryotic clade of Alveolata [9], are obligate parasites with an intracellular biotroph lifestyle (i.e. the host is maintained alive during the infection but eventually killed). A single infected host produces hundreds of small free-living dinospores that survive only for a few days outside the host and infect a wide range of taxa through multiple infection strategies, such as by actively penetrating into the host, entering through soft cuticles and wounds, or through intake by the host [4, 5, 10–12]. Syndiniales groups (SGs) I and II are the most common and diverse and are associated with various taxa that are potential zooplankton prey. SGs I and II dominate associations with zooplankton but have also been reported from fish eggs [13] as well as diverse protist hosts such as dinoflagellates, radiolarians, and ciliates [10, 14, 15]. SG III is generally less abundant, and some ASVs are correlated with phytoplankton hosts, particularly dinoflagellates and diatoms [16], although the sequences retrieved for SG III consist entirely of environmental sequences. SG IV includes two well-described genera: *Hematodinium*, which infects amphipods and decapod crustaceans, and *Syndinium*, a virulent parasite of copepods. Both have been associated with copepods, but the nature of the interactions remains unknown [4, 17, 18].

Parasitic spread depends on the interplay between host defence and parasite virulence [19, 20]. The spread is often shaped by environmental conditions, as most parasites are transmitted horizontally via free-living infective stages. Specific environmental factors, such as plankton blooms, might directly influence the spread of infections by increasing encounter rates and dinospore production [21–23]. For zooplankton, different transmission routes have been suggested [3]. The parasites enter their hosts through the direct intake of parasite dinospores from the water column; however, feeding on infected prey is another possibility [5, 24]. Therefore, the host feeding behaviour likely affects the transmission of the parasites. Additionally, host specificity may influence the ability of the parasite to control its hosts, as the behaviour of the host may change with proceeding infections.

Syndiniales associate and correlate with a variety of hosts, indicating potential infections across multiple host taxa [7, 10, 25]. The interactions may also relate to the host’s vertical position in the water column, since some parasites are adapted to specific habitats, such as low-oxygen environments [10, 26]. Nevertheless, the roles of abiotic factors, host feeding behavior, and potential host-specificity remain largely unexplored, leaving a substantial knowledge gap regarding marine ecosystem functioning.

In this study, we applied DNA metabarcoding of the 18S rRNA V4 gene region to amplify the gut content community of four selected zooplankton taxa and the water column to identify spatial and temporal variation in Syndiniales-zooplankton associations, the range of Syndiniales’ zooplankton hosts, and the drivers of these potential interactions. We investigated the Alveolata composition, particularly Syndiniales, in the water column and the associations with different zooplankton taxa along the Baltic Sea–Skagerrak environmental gradients of temperature, salinity, oxygen, and chlorophyll concentrations. Moreover, we explored the effect of zooplankton diet composition in shaping Syndiniales–zooplankton associations. Given the critical roles of zooplankton and Syndiniales in marine food webs, understanding their interactions is essential for predicting possible parasitic outbreaks and examining energy flow within the marine ecosystems.

## Materials and methods

### Sampling and sorting of the species

Water and zooplankton samples for 16S and 18S rRNA metabarcoding were collected at seven stations along the Baltic Sea-Skagerrak environmental gradient, with salinity ranging from 6.5 to 32.6, and were grouped into four sampling locations representing different sub-basins (Fig. S1 [27]) (Table S1). Station BY31 was sampled in June and August of 2017 and 2018 (see details in [28]). Samples from the other locations [Marine, Southern Baltic Sea (SBS), and Central Baltic Sea (CBS)] were collected in June 2019 and September 2020 during the Swedish Meteorological and Hydrological Institute (SMHI) monitoring cruises (see details in [29]). Environmental factors, including chlorophyll-*a* concentration, dissolved oxygen concentration, temperature, and salinity, were measured at each sampling location across the water column (Table S2) [30].

For water DNA metabarcoding, three replicate water samples were collected from each station and sampling depth when multiple depths were sampled (station BY31). Two liters of homogenized water from each depth stratum were filtered onboard using a peristaltic pump (Masterflex L/S) on 25 mm diameter polycarbonate filters with pore sizes of 20, 2.0, and 0.2 μm, which were later combined for DNA extraction [28, 29]. The filters were stored for further analysis at −20 °C and long-term at −80 °C. Zooplankton samples were collected by vertical hauls, using a 90-μm WP2 net from 25 m (Marine) or 30 m (SBS and CBS) depth to the surface, and in BY31 from the three depth strata 0–30 m, 30–60 m, and 60–90 m (Table S1). Zooplankton was preserved in 95% ethanol and stored at −20 °C until further analysis. The dominating zooplankton species were sorted for gut DNA metabarcoding, including the copepods *Acartia* spp. (including *A. bifilosa, A. longiremis,* and *A. tonsa*), *Centropages hamatus*, *Pseudocalanus* spp. (including *P. acuspes* and *P. elongatus)*, *Temora longicornis*, and the cladoceran *Evadne nordmannii*. From each sample, five individuals of each zooplankton species were hand-picked and rinsed in a 1% bleach bath to remove externally attached symbiont DNA, as described by [28]. We aimed for five replicates for each species and month; however, given that host species abundance varied over the season, the number of replicates varied per species (Table S1).

### DNA extraction, polymerase chain reaction, and sequencing

DNA extractions for water samples from BY31 were performed with the DNeasy Plant Mini Kit (Qiagen). For all other samples, the extractions were performed with a QIAmp DNA Micro Kit (Qiagen). The 16S libraries were processed as described in [31] (details in the Supplementary material) with a universal primer pair [32, 33]. For the 18S analysis, the V4 region of the 18S rRNA gene was amplified. The samples in BY31 were collected and processed as described in [28]. Briefly, the polymerase chain reaction (PCR) was performed in two rounds. A universal primer pair and a blocking primer were used to block amplification of calanoid copepod DNA (details in the Supplementary material) [34–36]. The 18S samples from all other locations than BY31 were collected and processed as described in [29]. Briefly, a nested PCR protocol was implemented by adding an initial pre-amplification step for the zooplankton samples with a calanoid copepod excluding primer pair [37]. The consecutive library preparations for all the samples were performed according to the best practices described in [33] (details in the Supplementary material).

### Bioinformatics and data analysis

Bioinformatic analyses were performed as described in [28]. Briefly, the cutadapt software [38] for primer removal, and the DADA2 package [39] was used for quality control, filtering, error rate modeling, and taxonomic assignment to amplicon sequence variants (ASVs) were performed in R [40]. To achieve adequate taxonomic resolution and to assign taxonomy to the 18S sequences, the PR2 database was utilized [41]. The 16S sequences were assigned to a custom-made database combining the SILVA 16S reference database [42] with the PhytoREF database [43] to assign both prokaryote and plastid sequences from eukaryote autotrophs.

To focus on the gut content of the hosts, we removed the crustacean reads from the dataset. After this removal, only samples with a read count of 500 or more were retained for analysis. Rarefaction curves of the total reads after quality filtration showed a steep increase and a plateau for all samples (Fig. S2), indicating sequencing effort adequacy. In total, 56 water and 110 host samples passed the quality filtration.

Data analyses and visualisation were conducted in R (4.2.3) using the tidyverse core packages [44], phyloseq [45], and circlize [46]. All statistical analyses were performed on the relative read abundance of each SG out of the total Syndiniales reads in each sample. The Shapiro-Wilk test was used to test for normality in the SGs relative read abundances across host and water samples among locations and water depth, and the Kruskal-Wallis test, followed by Dunn’s test with Bonferroni correction for pairwise comparison between locations. The frequency of occurrence of the Syndiniales across samples was calculated as the percentage of samples in which reads from a specific clade were observed.

To assess the role of environmental drivers on parasite relative read abundance, generalized linear models (GLM) were implemented. We tested the relationship between the different environmental variables and the relative read abundances of the SGs I to IV out of the total Syndiniales reads in the water and zooplankton hosts. Predictor variables included salinity, temperature, chlorophyll-*a*, and oxygen concentration averaged for each sampling event across the corresponding sampling depth and time point. Autocorrelation of the model residuals was tested with Durbin-Watson test, with no evidence of positive autocorrelation (DW = 1.93, *P* = 0.23). The predictor variables were included simultaneously as explanatory variables in the GLMs, assuming that residual variation was independent.

We also investigated the effect of host diet on the relative read abundances of SGs among zooplankton hosts. Diet composition was obtained from 16S and 18S rRNA metabarcoding data of photoautotrophic plankton and heterotrophic eukaryotes in the guts of the selected zooplankton, as described earlier [28, 29]. Diet composition for the 16S data was calculated as the average relative read abundance of cyanobacteria (separated by pico- and filamentous cyanobacteria), chlorophytes, diatoms, and other phytoplankton; see details in [47, 48], in each sampling location, host taxa, sampling date, and depth stratum. For the 18S diet composition analysis, the prey items were grouped based on taxonomy into Oligohymenophorea, Rhizaria, Dinophyceae, Spirotrichea, Opisthokonta, and Litostomatea. Random Forest regression trees were implemented using the randomForest package [49], which can handle nonlinear effects and complex interactions, while providing a ranking of variable importance in predicting the outcome. These models were used to analyze the effect of the relative read abundance of the diet taxonomic groups on the relative abundance of SG I, II, and IV as dependent variables. The relative abundances of each diet taxonomic group were used as predictor variables. For the 16S dataset, the number of trees was 500, and the number of variables tested at each split was 1. For the 18S dataset, the number of trees was 500, and the number of variables tested at each split was 2. Variable importance was assessed using the percentage increase in mean squared error (%IncMSE), calculated as %ΔMSE = (MSE_permuted – MSE_baseline)/MSE_baseline × 100. Higher values indicate substantially degraded model accuracy and that the predictor therefore contributed more strongly to the model. Spearman correlation analyses were performed with the corrplot package [50] and Bonferroni correction to account for multiple testing, to provide an overview of pairwise linear associations, and to identify potential multicollinearity. Correlation analysis was also used to assess relationships between the SGs associated with zooplankton and diet taxa [51].

## Results

### Overview of reads and ASVs

Crustaceans accounted for about 80% on average of the reads in the zooplankton samples. After quality filtration, 56 water samples yielded 5.68 M reads, and 110 zooplankton samples yielded 3.26 M reads. Among the Alveolata, Dinophyceae contained the highest number of ASVs (674 ASVs) across all the samples and locations, ranging from 86 ASVs in CBS to 247 ASVs in BY31. This was followed by Syndiniales, which contained a total of 369 ASVs. Of these, one-third belonged to SG I (123 ASVs), while SG II was the most diverse group, represented by 203 ASVs found both in the water samples and in association with hosts across all locations (Table S3). The average 16S relative read abundance in the different zooplankton taxa indicated that cyanobacteria dominated the diet in all Baltic Sea locations, while diatoms dominated in the Marine location (Fig. S3).

### Relative abundance, supergroups, and classes

**Figure 1:**
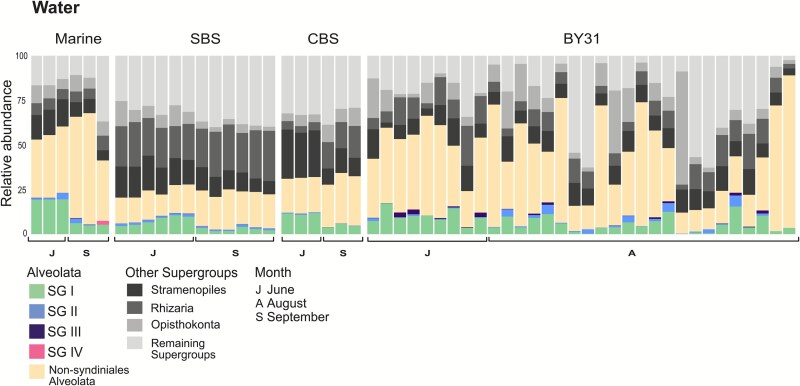
Relative read abundance of Alveolata and non-Alveolata eukaryotic (other) supergroups in the water column at the different sampling locations and sampling months. Syndiniales reads represent, on average, 9.4% of the total reads across the locations and sampling months. Bars represent individual replicates.

In the water samples, Alveolata dominated the eukaryotic community after crustacean reads were removed relative read abundance, contributing about 60% of reads the m Marine location and between 23% and 40% in the other locations (Fig. 1). Other abundant supergroups in the water were Stramenopiles, Rhizaria, and Opisthokonta, but on average in lower percentages than the Alveolata. Syndiniales represented 9.4% of the total reads in the water samples, with the relative abundance of SG I being higher in the Marine location in comparison to other locations (Kruskal-Wallis, chi-sq = 18.15, df = 3, *P* < 0.01), while SG II abundance differed significantly between locations (chi-sq = 75.15, df = 3, *P* < 0.01). For both SG II and III, the abundance differed between the depth strata in BY31 (chi-sq = 29.72, df = 2, *P* < 0.01, and chi-sq = 21.74, df = 2, *P* < 0.01, respectively). SG IV relative abundances in the water were low (< 4%) across all locations and only present in 19 out of 56 water samples (chi-sq = 3.06, df = 3, *P* = 0.38) (Fig. 1).

**Figure 2:**
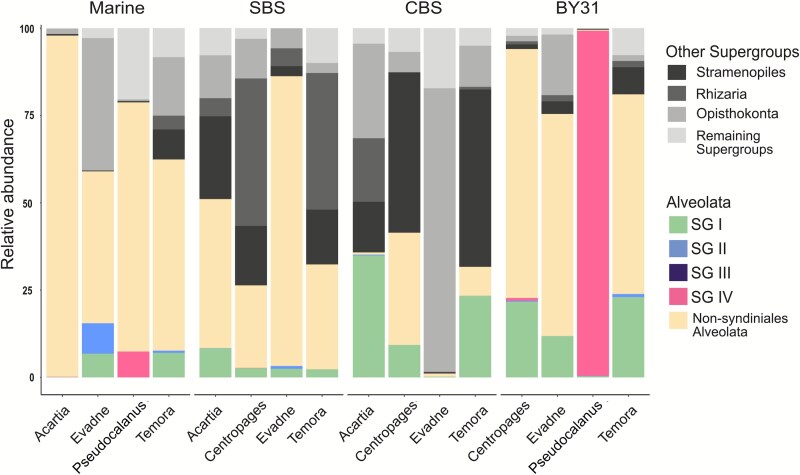
Relative read abundance of Alveolata and non-Alveolata eukaryotic (other) taxa associated with the different host zooplankton taxa across the sampling stations. Each bar shows the average relative abundance for each host taxon across all samples.

The proportion of Alveolata reads associated with zooplankton hosts was variable between species and sampling months, ranging from null to almost 100% (Fig. S4). Similarly, the proportions of other supergroups varied across zooplankton taxa and between the locations (Fig. S4). Among Alveolata reads, the Syndiniales represented on average more than 30% of the relative read abundance in host samples across sampling locations, with varying proportions of the different SGs in the zooplankton taxa (Fig. 2).

### Syndiniales clades

**Figure 3:**
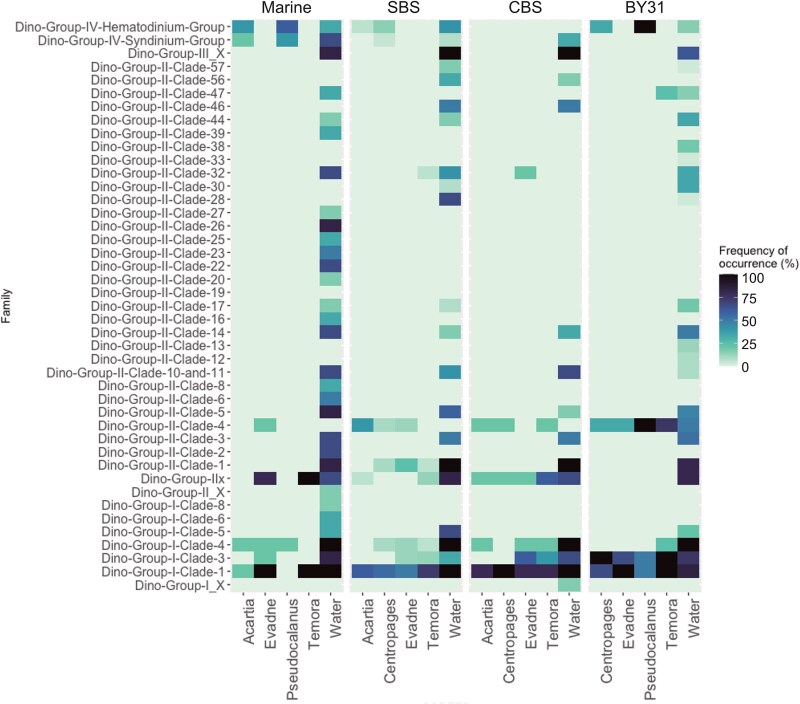
Heatmap showing the frequency of occurrence of Syndiniales clades in the water column and associated with zooplankton hosts at each sampling location. The intensity of the colour indicates the average percentage of the samples in which a clade is present across all samples.

We retrieved sequences taxonomically assigned to seven clades of SG I, 32 clades of SG II, and the genera *Hematodinium* and *Syndinium* of SG IV across all samples. SG III was present in most of the water samples across the locations, but the taxonomic assignment of SG III only reached the group level. No associations between the hosts and SG III were observed. The water samples had the highest number of clades, particularly in the Marine location, with 31 out of 43 clades identified (Fig. 3). The frequency of occurrence varied across clades, with only a few clades present in almost all samples. The clades associated with zooplankton belonged mainly to SG I and SG IV. Only five out of 32 SG II clades were associated with zooplankton hosts, and often with a low frequency of occurrence. SG IV *Hematodinium* occurred more frequently in host *Pseudocalanus* spp. than in the water (Fig. 3). Some of the most frequent clades in the water samples were also observed in the host samples, with varying frequencies depending on the host. Notably, SG II C-4 was the only clade detected across multiple hosts but did not occur in water samples, except in BY31 (Fig. 3).

### Host associations of Syndiniales groups

**Figure 4:**
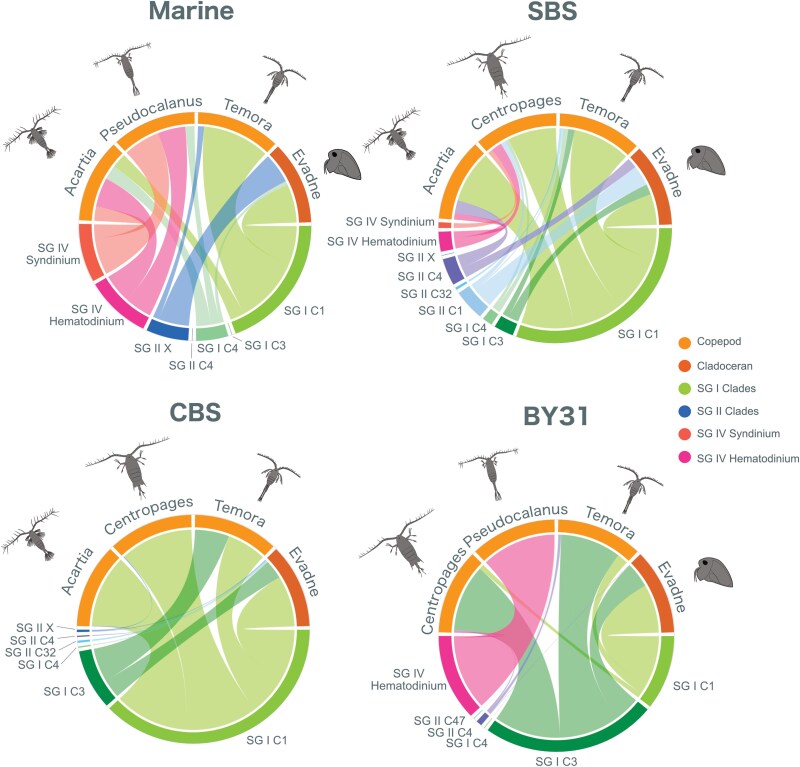
Host-specific interactions of Syndiniales groups (SG) I, II, and IV (I-IV) and their crustacean zooplankton hosts at each sampling location. SG clades are presented with colour shades at the lower part of each circle plot, and the associated host taxa are presented in the top part of the circle. The width of each link represents the relative read abundance of each Syndiniales clade associated with the host taxa. SG I consists of three clades (green shades); SG II of five clades (blue shades), and SG IV of the genera *Hematodinium* and SG IV *Syndinium* (red shades).

Host-specific interactions with SGs showed that *Pseudocalanus* spp. were primarily associated with SG IV in the Marine location (Fig. 4), contributing to about 17% of the total Alveolata reads (Fig. 2). Out of the Syndiniales reads in *Pseudocalanus* spp., SG IV *Hematodinium* and SG IV *Syndinium* represented 38% and 50%, respectively (Fig. 4). At BY31, *Pseudocalanu*s spp. associations dominated with SG IV *Hematodinium*, which accounted for almost 100% of all Alveolata reads. The copepods *Acartia* spp. were mainly associated with SG I and SG II in the CBS and SBS locations, whereas in the Marine location, they were mostly associated with SG IV *Hematodinium* (40%) and SG IV *Syndinium* (20%) (Fig. 4). *Centropages* was mainly associated with SG I and in low proportion with SG IV (8% of the total Alveolata reads). For *T. longicornis*, SG I contributed to more than 90% of the SG reads in all locations, while *T. longicornis* was not associated with SG IV in any location. The cladoceran *E. nordmannii* was associated with SG I and II (average SG reads relative to the total 18S reads = 10.8%) in similar proportions as in the water samples (average SG reads relative to the total 18*S* reads = 9.4%) (Fig. 1).

### Environmental effects on SG relative abundance

The environmental variables were relatively similar in the surface layer across locations and sampling months, except for salinity, which was higher in the Marine location. Oxygen concentration decreased with increasing depth in BY31 (Table S2). The relative abundances of the individual SGs, calculated relative to total Syndiniales reads, were explained by different environmental factors across all samples (Table S4). SG I relative abundance was negatively related to temperature (incidence rate ratio [IRR] = 0.94) and positively to oxygen (IRR = 1.26), although effect sizes (IRR) were low. SG II relative abundance was related positively to oxygen (IRR = 2.18). SG III showed a positive relation to chlorophyll-*a* (IRR = 8.59) and a negative relation to temperature (IRR = 0.60). SG IV was negatively associated with oxygen concentration (IRR = 0.20) (Table S4). Notably, SG IV was associated with zooplankton hosts only at salinities above 7.7. In the lowest salinity locations of CBS and BY31 surface strata, SG IV was detected in the water column (Table S2, Fig. 4).

### Correlations between Syndiniales groups and host diet composition

**Figure 5:**
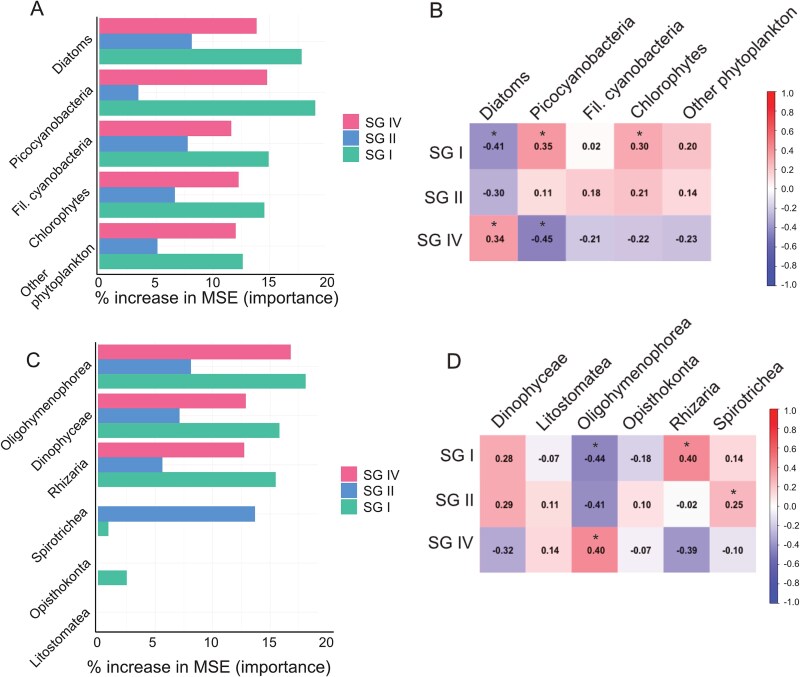
Relationships between Syndiniales groups (SGs) and phytoplankton prey across all zooplankton host taxa and locations. A) Important prey taxa selected by random forest based on the percentage increase in mean squared error (% increase in MSE) classificationacross all SGs for prey items identified by 16S. Higher values indicate that permuting a variable worsened model performance and, therefore, the predictor contributed more strongly to the model. Prey taxa are ordered based on their contributions from top (most important) to bottom (least important) B) Spearman’s rho correlation of phytoplankton diet composition (16S) and relative read abundance of SGs across all zooplankton hosts. C) Random forest based on percentage increase in mean squared error (%IncMSE) classification across all SGs for prey items identified by 18S. Higher values indicate that permuting a variable worsened model performance and, therefore, the predictor contributed more strongly to the model. Prey items are ordered based on their contributions from top (most important) to bottom (least important) for each Syndiniales group. Litostomatea showed no importance to any of the SG relative read abundances. D) Spearman’s rho correlation of the heterotrophic prey taxa composition (18S) and SGs relative read abundance across all zooplankton hosts. All significant (16S and 18S) correlations (*P* < 0. 5) are marked with an asterisk symbol (^*^) using a 95% confidence level; *P*-values were Bonferroni adjusted for multiple testing. Prey items are ordered based on the overall performance from left to right. The “other phytoplankton” include Ochrophytes (Chromulinales, Dictyochophyceae, Eustigmatales, and unidentified Ochrophyta), haptophytes (Prymnesiales), and cryptophytes (Pyrenomonadales).

The autotrophic diet composition (identified with 16S) explained varying degrees of Syndiniales relative abundance, with random forest analysis showing it accounted for 24.4% of the variance for SG I and 21.4 % for SG IV. No variance in SG II abundance was explained by the model. Diatoms had the highest explanatory power in the random forest tree classification, followed by picocyanobacteria and filamentous cyanobacteria (Fig. 5A). The correlation analysis supported the random forest outcome, showing significant but opposite correlations of diatoms and picocyanobacteria with the relative abundances of SG I and IV (Fig. 5B). Random forest analysis of the 18S showed that 17% of the variance in SG I was explained by the diet. For SG IV the variance explained was 10.2%, and 5.9% for SG II. The highest explanatory power for the 18S diet components were Oligohymenophorea, followed by Dinophyceae and Rhizaria (Fig. 5C). Spirotrichea emerged as an important predictor for SG II. The correlation analysis revealed similar patterns, showing significant correlations for Oligohymenophorea with SGs I and IV, Rhizaria with SG I, and Spirotrichea with SG II (Fig. 5D). The nature of the correlations varied among SGs and prey taxa, with negative correlations indicating exclusion and positive correlations indicating co-occurrence of SGs and prey taxa.

## Discussion

Current research highlights the importance of parasitism in shaping plankton population dynamics, with much attention given to parasites infecting phytoplankton blooms [14, 51]. In this study, we investigated Syndiniales–zooplankton interactions and the key drivers of their dynamics, such as abiotic conditions and the diet composition of zooplankton taxa. We found that Syndiniales were prevalent, with higher ASV richness at higher salinity. Syndiniales were associated with diverse zooplankton hosts, suggesting generalist associations for SG I and II, and more zooplankton taxa–specific associations for SG IV. We showed that the interactions between Syndiniales and zooplankton correlate with diet composition and environmental variation, with varying effects among SGs. Our findings emphasize the key environmental drivers of Syndiniales interactions and the important role of zooplankton–Syndiniales associations in structuring plankton communities.

Our results show that Alveolata dominate the protist relative read abundance of the total 18S reads in the water column. Syndiniales reads represent, on average, one-third of the Alveolata reads in the zooplankton host samples, varying between 8% and 99%. However, the high proportion of Alveolata and Syndiniales may be overrepresented due to their high 18S rRNA gene copy number [52, 53]. While a high gene copy number needs to be taken into consideration, high Syndiniales relative read abundance associated with zooplankton may also indicate active reproduction within the host, albeit this supposition needs to be confirmed with an experimental setup or microscopy. Further, the lack of existing reference sequences and the taxonomic resolution in the databases might hinder the identification of all taxa present in the samples; as for many sequences, the taxonomic assignment reached only the group level. Among Syndiniales, SGs I and II were most abundant across the environmental gradients and associated with the zooplankton. SG IV was most abundant in association with some copepod hosts and reached low relative read abundance in the water column, but was frequently observed. The number of ASVs was substantially lower in brackish water locations than in locations with higher salinity, which supports findings of reduced taxa diversity in brackish water [32, 54]. Our results are in line with observations from other coastal and oceanic systems showing that SG I and II are prevalent and diverse in the water column, with ecological niches related to salinity or depth [6, 16, 26].

### Syndiniales-zooplankton interactions

Our results revealed distinct associations between Syndiniales clades and zooplankton host taxa. While a high diversity of Syndiniales was identified in the water column, only a few clades were associated with the zooplankton hosts across all locations. This pattern may not only be related to lower read counts in zooplankton samples in comparison to the water samples, but may also indicate some specificity. Syndiniales are presumably transmitted into zooplankton through prey items, either from direct dinospore intake or by secondary intake through consuming infected prey, albeit penetration through the host’s exoskeleton cannot be excluded [4, 24]. SG I was associated with all zooplankton taxa. SG I Clades 1, 3, and 4 dominated the host associations and were also most frequent in the water column, consistent with multiple possible transmission routes. SG II was relatively low in abundance in both the water and all zooplankton hosts. The *Amoebophyra* species complex, of which some sequences have been assigned to SG II C-4, is known to infect dinoflagellates [10, 14]. It was observed in association with all zooplankton taxa of our samples, but in the water column only in BY31. Therefore, the intake of this clade most likely occurs through infected prey that possibly host the different life stages of the parasite [55], rather than direct consumption of the dinospores from the water. Our results support previous observations that SG I and II are associated with a wide host range, including various metazoans and protists [10, 15, 56], suggesting that generalist associations with various taxa are likely common for SG I and SG II.

In contrast, SG IV was primarily associated with the copepods *Pseudocalanus* spp. at the Marine location, and in the deep-water strata of BY31. In the marine location, *Pseudocalanus* spp. were linked to both *Hematodinium* and *Syndinium*, although *Hematodinium* predominated in interactions at BY31. SG IV *Syndinium* is a well-known, highly virulent parasite genus in copepod species, causing mortality [18]. The phylogenetic sister taxon *Hematodinium* is a common parasite in crustaceans, leading to significant losses in langoustine and crab fisheries [57], but its effects on copepods are not well understood. The transmission route of *Hematodinium* into the copepods remains unknown, but the high abundance of reads associated with *Pseudocalanus* spp. in our samples, despite low read abundances in the water column, might be an indicator of reproduction within the zooplankton hosts. While a parasitic relationship with copepod hosts requires confirmation through microscopy and experimental analyses, our results imply that SG IV *Hematodinium* exhibits a more specific host range, primarily associated with copepods in the pelagic environment.

### Environmental effects on Syndiniales groups

SG IV relative abundance was significantly higher under low-oxygen concentrations. This pattern is likely driven by *Hematodinium* infecting *Pseudocalanus* spp. in the deep waters at BY31. At this station, the oxygen concentration declines rapidly below 60 m depth to levels of <2 mg L^−1^. Syndiniales have adapted strategies to function under oxygen-limited conditions [25, 58, 59], and SG IV in particular was observed in the *Pseudocalanus* spp. samples from low-oxygenated habitats. Our results show for the first time, to our knowledge, that the association between SG IV and copepods increases in low-oxygen environments. *Pseudocalanus* spp. prefer higher salinities, and is therefore forced to live below the halocline in the Baltic Sea [60, 61]. These environments are often hypoxic, which may add stress on the copepods, possibly lowering their fitness and thus making them more vulnerable to parasitic infections. The extent to which the association with *Hematodinium* contributes to the declining biomass of *Pseudocalanus* spp. [62, 63] remains unexplored. However, given *Hematodinium'*s high relative read abundance in *Pseudocalanus* spp., and parasitic relationship with other crustaceans, it potentially affects copepod fitness. The spread of low-oxygenated zones in coastal environments with rising temperatures [64] will likely extend environmental niches favorable to this interaction, causing an additional source of zooplankton mortality. Increased copepod mortality likely affects energy transfer to fish, as *Pseudocalanus* spp. are important prey items [63], and instead redirects organic matter into the microbial loop [65], altering carbon cycling and the overall ecosystem productivity.

In comparison to SG IV, SG I and II were positively related to oxygen concentration, which is confirmed on a larger scale, as these groups are often abundant in surface waters [10, 66]. However, the effect size of oxygen was low, similar to the negative but significant effect of temperature on SG I. SG III was negatively related to temperature. Different and opposing relationships between abiotic factors and SGs may indicate habitat or depth-specific niches of the SGs, supporting previous findings of limited effects of temperature and salinity on Syndiniales abundances [26]. Moreover, the proportion of SGs did not differ between sampling seasons, which supports the notion that Syndiniales are present year-round without any strong seasonal patterns related to their associations. Environmental conditions, particularly ecosystem productivity indicated by chlorophyll concentration, had a positive effect on SG III, suggesting that this group mainly interacts with phytoplankton taxa. These findings indicate that Syndiniales can be highly abundant in coastal and brackish water habitats, and that their ecological niche preferences vary among SGs, allowing them to interact with a wide range of organisms.

### Diet composition shapes Syndiniales-zooplankton interactions

The feeding strategies of the zooplankton species in our study have been distinguished, including filter feeders and ambush-feeders [29]. Given that copepods generally feed less efficiently on prey smaller than 20 μm in size, we assume that Syndiniales intake is primarily via consumption of infected prey items. Random forest results identified different dietary components as explanatory variables for SG relative abundances in the hosts. Correlation analyses confirmed that the prey components were significantly related to SGs I, II, and IV, albeit with varying effects. The negative correlations with SG I, specifically for diatom and Oligohymenophorea, suggest that higher abundances of these prey components are associated with reduced intake of SG I. Negative diatom–Syndiniales relations follow the Syndiniales–eukaryotic community interactions at a larger scale [7, 16], indicative of limited interactions of diatoms with SG I. However, SG I was positively associated with Dinophyceae, suggesting transmission via infected prey items, as SG I is reported to associate with dinoflagellates [7]. Rhizaria showed a significant positive correlation with SG I, which was also the most abundant SG in the zooplankton that was associated with Rhizaria (*C. hamatus* and *T. longicornis* in SBS). These findings support the importance of the diet and the possible route of intake through prey components that are associated with the Syndiniales.

SG II was significantly associated with only Spirotrichea, supporting that this parasitic group mainly associates with heterotrophic protists [7, 14]. Opposite to SG I, SG IV occurrence was positively related to diatoms and Oligohymenophorea, but negatively to the remaining prey components. SG IV read abundance, along with diatom and Oligohymenophorea prey abundance, was highest in *Pseudocalanus* spp., which actively select their prey [67]. This indicates the association of SG IV and diatoms, similar to the co-occurrence reported in other studies [7, 24, 25], although SG IV infection of diatoms has not been confirmed. Picocyanobacteria, an important prey component of zooplankton taxa [31], were negatively related to SG IV. To our knowledge, Syndiniales and picocyanobacteria interactions have not been reported, as the prokaryotic community remains to be included in network analysis. In contrast to copepods, the filter-feeding cladoceran *E. nordmannii* interacted with the SGs that were most abundant in the water column, which supports its unselective feeding behaviour [68]. Our findings suggest that zooplankton intake of SG I occurs indirectly through infected prey, while the transmission route of SG IV remains uncertain, as the consumption of infected diatoms and Oligohymenophorea must be further investigated.

## Conclusions

Our results show that Syndiniales are associated with zooplankton across the brackish and marine environmental gradient. Our results suggest that zooplankton are most likely not the final hosts for SG I and II, and that their intake is indirect through infected prey, albeit direct infection on crustacean zooplankton with these groups cannot be excluded. However, copepods are likely final hosts for SG IV, given the sheer read abundance associated with copepods and the documented parasitic infections of certain taxa [18]. Moreover, we did not detect any host species-specificity on SGs I and II, supporting that Syndiniales are generalist parasites [7, 15, 16], an infection strategy that is beneficial in highly dynamic plankton systems where host organisms exhibit strong abundance fluctuations. Our findings further substantiate the role of environmental conditions, particularly low oxygen concentrations, in shaping Syndiniales interactions with zooplankton, driven by the shared niche of both host and parasite. Establishing the association rates and the ecological roles of parasites within a population will provide further knowledge of how parasite infections influence the food web and carbon fluxes in pelagic systems.

## Supplementary Material

Supplementary_material_final_ycaf248

Supplementary_Table_4_ycaf248

## Data Availability

The raw sequence data can be found on the European Nucleotide Archive (ENA) website, under the project numbers: PRJEB39191 and PRJEB52087. The environmental data are available at the Swedish national archive for oceanographic data (https://sharkweb.smhi.se/) [30]. The original datasets, the environmental data, and an RMarkdown document for the data analysis are provided in online repositories: https://github.com/neeahanstrom/Syndiniales-zooplankton-interactions; https://doi.org/10.5281/zenodo.20309621.
