# Supplementary material for: Diet composition and environmental niche drive parasitic Syndiniales interactions with crustacean zooplankton
Source: ISME Commun. 2025 Dec 24;6(1):ycaf248. doi: 10.1093/ismeco/ycaf248 (PMC13245725; doi:10.1093/ismeco/ycaf248)
Supplement: Supplementary_material_final_ycaf248 [file supplementary_material_final_ycaf248.pdf]

Diet composition and environmental niche drive parasitic Syndiniales interactions with crustacean zooplankton

Neea Hanström, Kinlan M. G. Jan, Baptiste Serandour, Tianshuo Xu, Monika Winder

Supplementary material

Materials and methods: Detailed description

DNA extraction, PCR, and sequencing

The DNA extractions from BY31 water samples were performed using the DNeasy Plant Mini Kit (Qiagen). For all other samples, the extractions were performed with the QIAmp DNA Micro Kit (Qiagen). The 16S samples were processed as described by [1]. For the 16S analysis, the V3-V4 region of the 16S rRNA gene was amplified with the universal primer pair 341F (CCTACGGGNGGCWGCAG) and 805R (GACTACHVGGGTATCTAATCC) targeting prokaryotes and the plastids of eukaryotic phototrophs [2,3].

For the 18S analysis, the V4 region of the 18S rRNA gene was PCR-amplified for the samples collected in BY31 as described in [4]. The universal primer pair 528F (GCGGTAATTCCAGCTCCAA) and 706R (AATCCRAGAATTTCACCTCT) [5,6] was used. A peptide nucleic acid (PNA) was designed (N-AAGAATTTCACCTCTGTCG-Lys-C) by modifying the 18s_cal_706r primer from [6,7] to block amplification of calanoid copepod DNA [4].

The PCR for BY31 samples was performed in two steps as described by [4]. The first PCR conditions were as follows: 98 °C for 2 min followed by 25 cycles of 98°C for 20s, 63°C (16S), or 54°C (18S) annealing for 20s, 72 °C for 15s, and a final elongation step of 2 min at 72 °C. The second PCR amplification was performed in 28 µL volume, and reactions contained 14 µL of KAPA HiFi HotStart ReadyMix, 1 µL Handle1 (index_forward)-Adapter1 (10 µM), 1 ll Handle2 (index_reverse)-Adapter2 (10 µM), and 12 µL of cleaned PCR product. The thermocycling conditions were: 98 °C for 2 min followed by 10 cycles of 98°C for 20 s, 62°C for 30 s, 72°C for 30 s, and a final elongation step of 2 min at 72°C.

The 18S samples collected from all locations other than BY31 were processed as described in [8]. A nested PCR protocol was implemented by adding an initial pre-amplification step for the zooplankton samples, amplifying all but calanoid copepods. The primer pair used (non-copepod 18SF2*:* AGCAGGCGGHAAATTRCCAATCY and non-copepod 18SR2*:* CCGTGTTGAGTCAAATTAAGCCG) [9] amplifies an extended segment of the V4 region of the 18S gene, binding universally, but excludes calanoid copepods. For the PCR, we used 10 µL of KAPA HiFi HotStart ReadyMix (Roche, KAPA Biosystems), 10 pmol of each primer, 6 µL of molecular-grade water, and 2 µL of extracted DNA template with varying concentrations. The thermocycler conditions consisted of 2 min of initial denaturation at 98°C followed by 5 cycles of denaturation of 20s at 98°C, an annealing of 20s at 68°C, and an elongation for 15s at 72°C, followed by 10 cycles with denaturation of 20s at 62°C, and an elongation for 15s at 72°C. The final elongation was completed with 60s at 72°C. The consecutive library preparations were performed according to the best practices described by [3]. Similarly to the samples from BY31, the library preparation consisted of two steps, where the first one amplified the targeted gene region and the second one binded primers containing the sample specific indexes. PCR products were purified using XP magnetic beads (Agencourt AMPure XP, Beckman Coulter) between the two steps and at the end of the library preparations [4,8].

References

1. Novotny A, Zamora-Terol S, Winder M. DNA metabarcoding reveals trophic niche diversity of micro and mesozooplankton species. *Proceedings of the Royal Society B: Biological Sciences* 2021;**288**, DOI: 10.1098/rspb.2021.0908.
2. Herlemann DPR, Labrenz M, Jürgens K, Bertilsson S, Waniek JJ, Andersson AF. Transitions in bacterial communities along the 2000 km salinity gradient of the Baltic Sea. *ISME Journal* 2011;**5**, DOI: 10.1038/ismej.2011.41.
3. Hu YOO, Karlson B, Charvet S, Andersson AF. Diversity of pico- to mesoplankton along the 2000 km salinity gradient of the Baltic Sea. *Front Microbiol* 2016;**7**, DOI: 10.3389/fmicb.2016.00679.
4. Zamora-Terol S, Novotny A, Winder M. Reconstructing marine plankton food web interactions using DNA metabarcoding. *Mol Ecol* 2020;**29**, DOI: 10.1111/mec.15555.
5. Elwood HJ, Olsen GJ, Sogin ML. The small-subunit ribosomal RNA gene sequences from the hypotrichous ciliates Oxytricha nova and Stylonychia pustulata. *Mol Biol Evol* 1985;**2**, DOI: 10.1093/oxfordjournals.molbev.a040362.
6. Ho TW, Hwang JS, Cheung MK, Kwan HS, Wong CK. DNA-based study of the diet of the marine calanoid copepod Calanus sinicus. *J Exp Mar Biol Ecol* 2017;**494**, DOI: 10.1016/j.jembe.2017.04.004.

7. Yi X, Huang Y, Zhuang Y, Chen H, Yang F, Wang W *et al.* In situ diet of the copepod Calanus sinicus in coastal waters of the South Yellow Sea and the Bohai Sea. *Acta Oceanologica Sinica* 2017;**36**, DOI: 10.1007/s13131-017-0974-6.
8. Serandour B, Jan KMG, Novotny A, Winder M. Opportunistic vs selective feeding strategies of zooplankton under changing environmental conditions. *J Plankton Res* 2023, DOI: 10.1093/plankt/fbad007.
9. Guo Z, Liu S, Hu S, Li T, Huang Y, Liu G *et al*. Prevalent Ciliate Symbiosis on Copepods: High Genetic Diversity and Wide Distribution Detected Using Small Subunit Ribosomal RNA Gene. *PLoS One* 2012;**7**, DOI: 10.1371/journal.pone.0044847.

Supplementary Figures

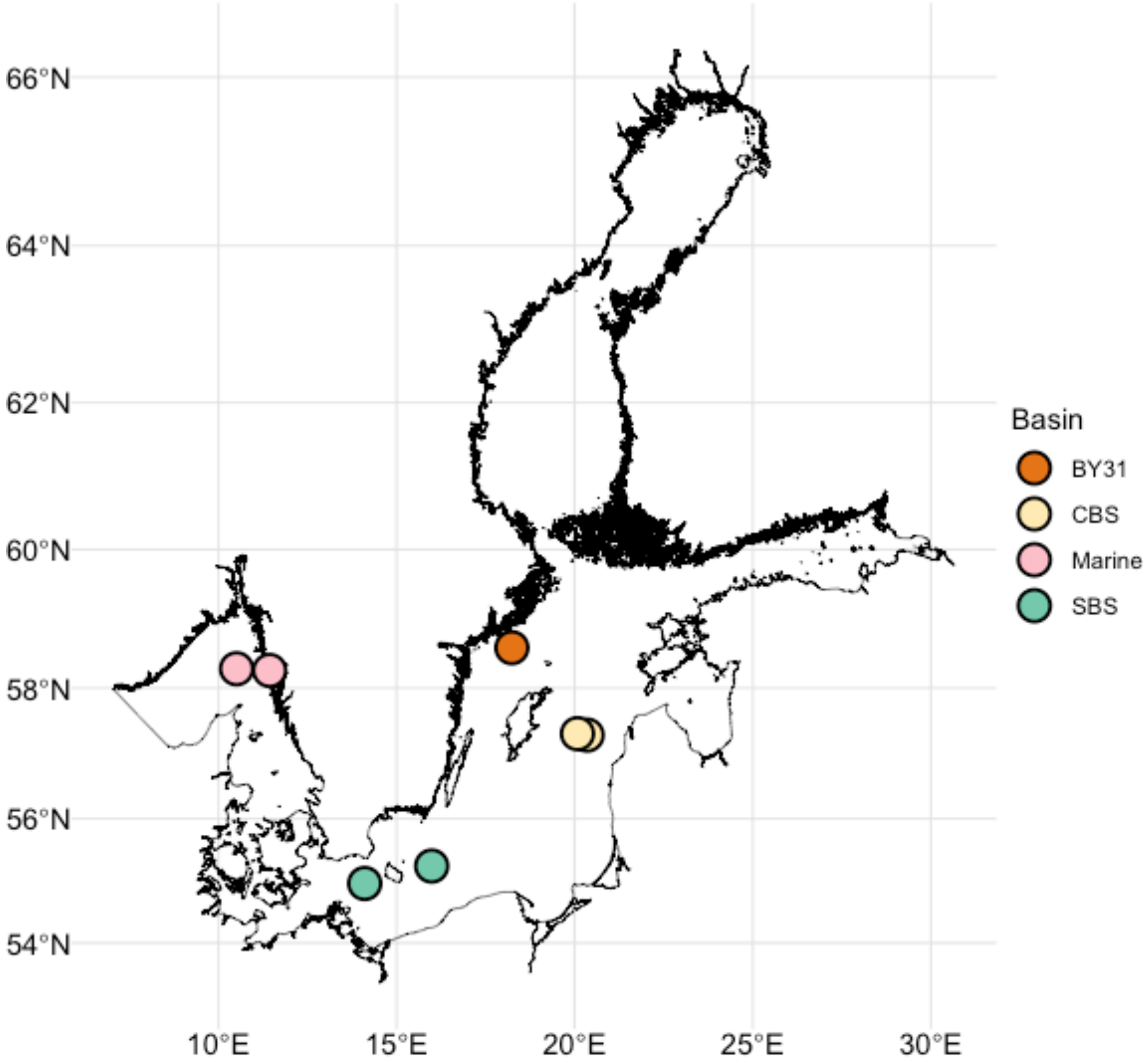

Supplementary Figure 1. Map of the Baltic Sea showing the four sampling locations: BY31, Central Baltic Sea (CBS), Southern Baltic Sea (SBS), and Marine. Each location includes two sampling stations, except BY31. Marine (pink) includes Å17 and Släggö, SBS (green) includes BY2 and BY5, and CBS (yellow) includes BY15 and BY16. BY31 is presented in brown. Coordinate data for the map creation were retrieved from ICES. (2005)[68].

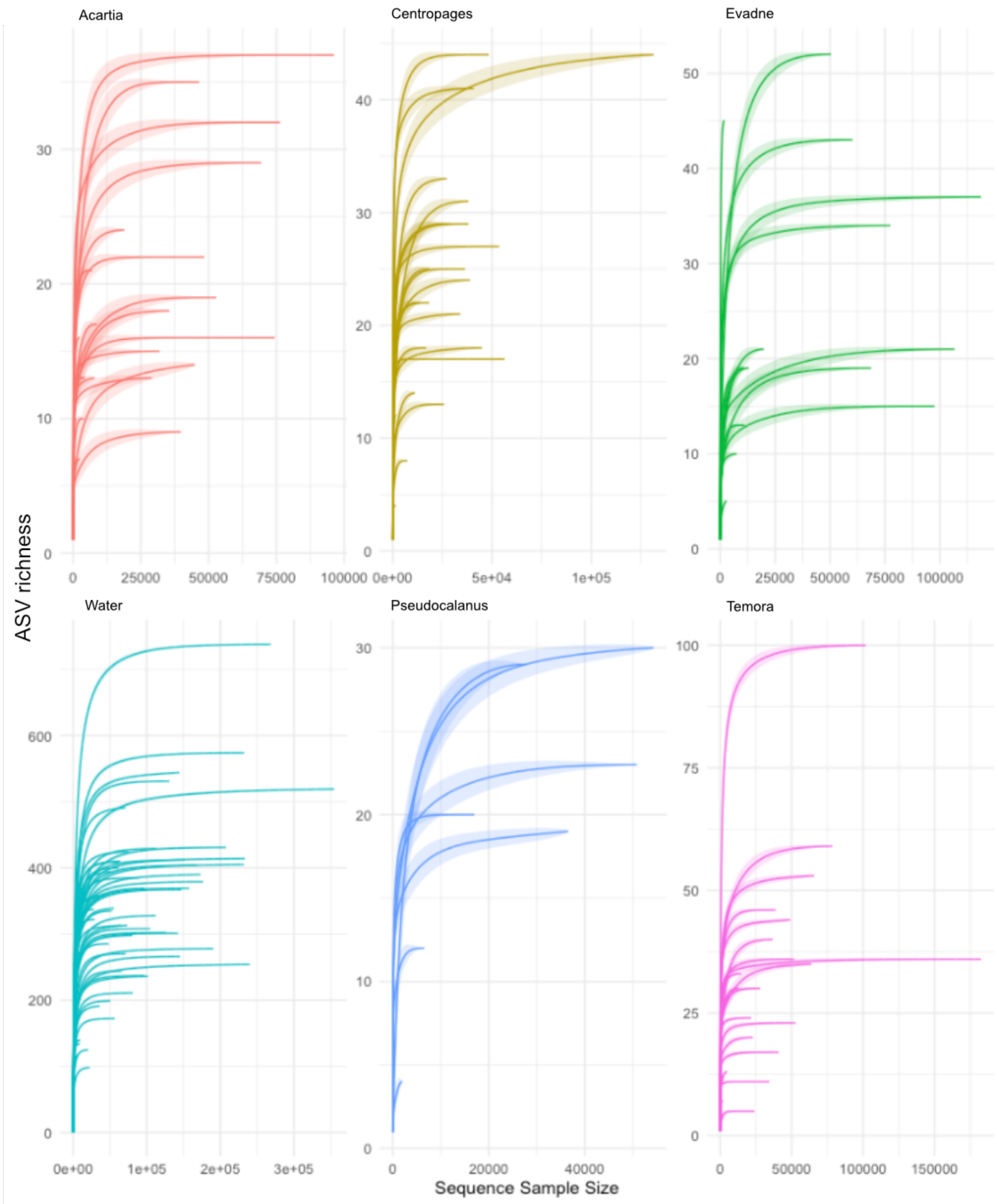

Supplementary Figure 2. Rarefaction curves for the plankton community across all samples, presented by each host taxon and the water column. The X-axis represents the number of sequencing reads, and the y-axis shows the species richness in the observed number of amplicon sequence variants (ASVs) in each sample. The plateau in each curve indicates that the majority of the species diversity has been captured.

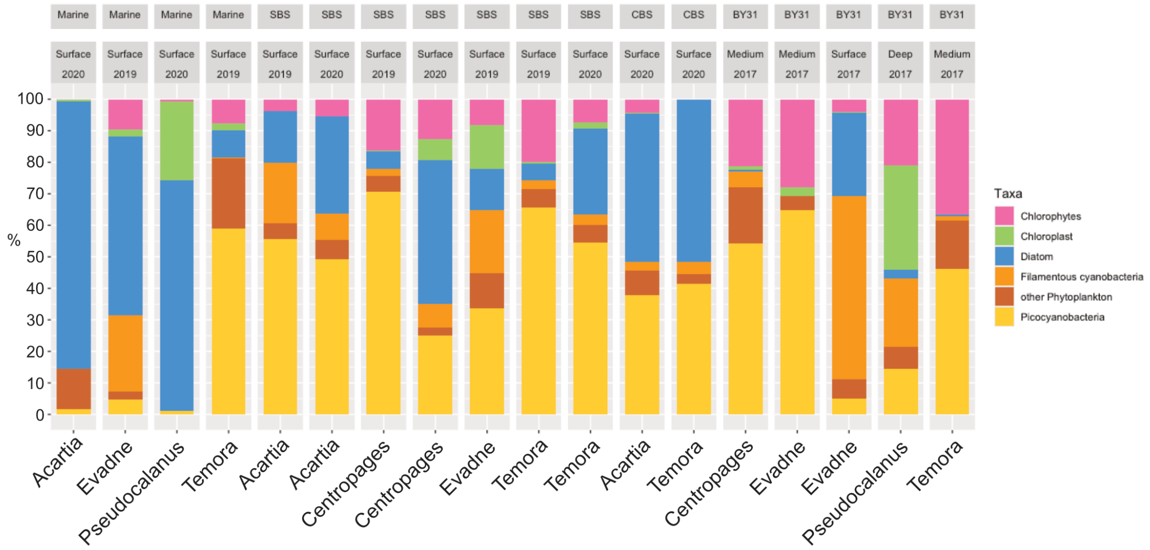

Supplementary Figure 3. Overview of the average relative read abundance of the 16S diet component in the zooplankton taxa in each sampling location, depth and year. Surface samples were collected at 0-30m depth, medium at 30-60m depth, and deep at 60-100m.

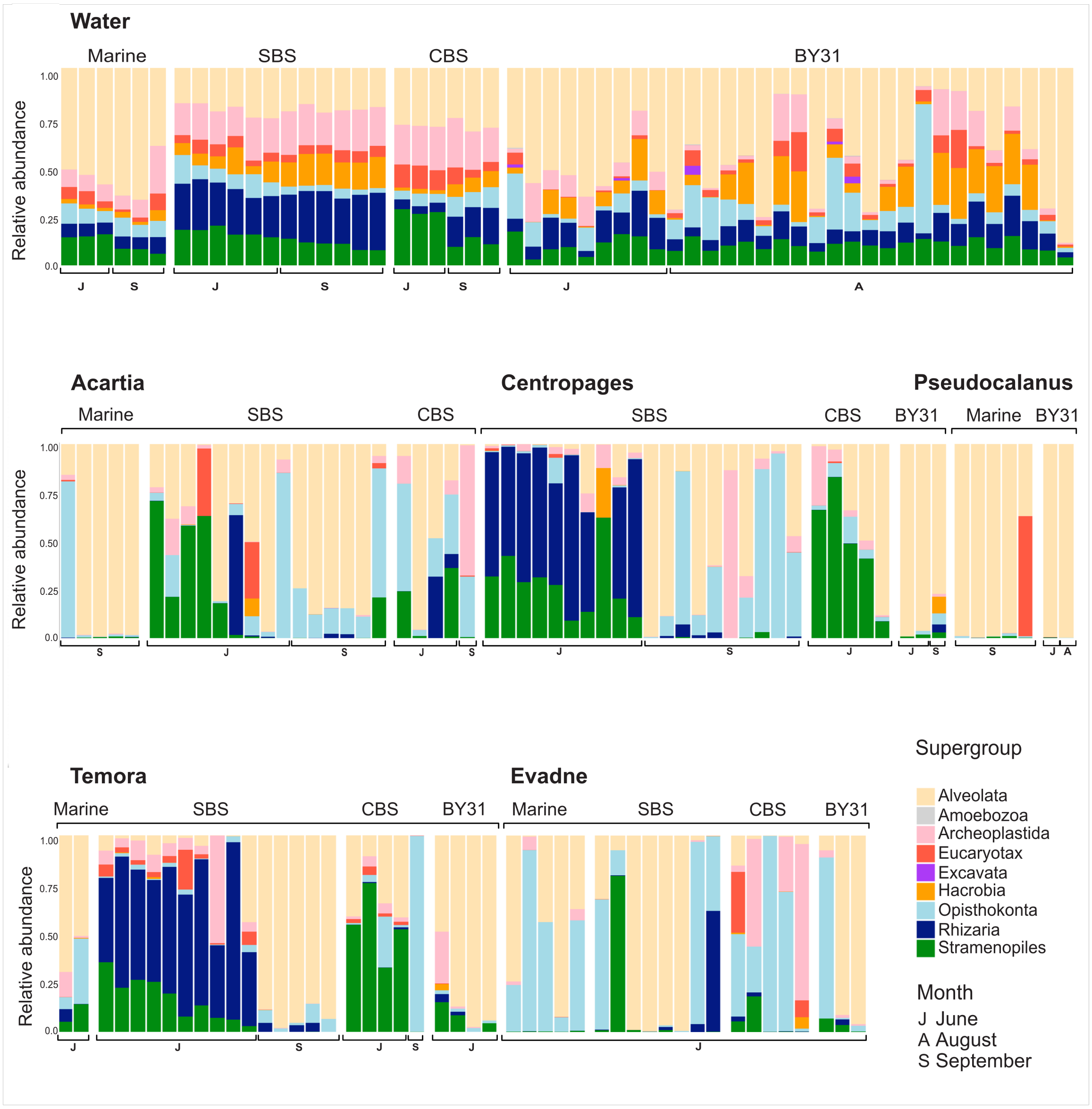

Supplementary Figure 4. Relative abundance of eukaryotic plankton composition based on 18*S* rRNA gene sequencing. The figure shows the composition of eukaryotic supergroups in both water column samples and zooplankton hosts at different locations. Reads corresponding to Crustacea have been excluded. Samples are organized by sampling month: J = June, S = September, and A = August.

Supplementary Tables

Supplementary Table 1. The sampling stations, zooplankton collection depths, and number of zooplankton host samples at the four sampling locations. Each zooplankton sample includes five individuals. The number on the zooplankton samples indicates the number of samples that passed the quality filtration and were analyzed from the different locations. Locations are BY31, Marine, Southern Baltic Sea (SBS), and Central Baltic Sea (CBS). The zooplankton collection depths are marked with letters (S = surface, M = medium, and D = deep).

	Marine	SBS	CBS	BY31
Sample type	Sampling dates			
Acartia spp.	9/2020	6/2019; 9/2020	6/2019; 9/2020	
Centropages hamatus		6/2019; 9/2020	6/2019; 9/2020	6/2017; 8/2017
Pseudocalanus spp.	9/2020			6/2017; 8/2017
Temora longicornis	6/2019	6/2019; 9/2020	6/2019; 9/2020	6/2017
Evadne normannii	6/2019	6/2019	6/2019	6/2017
Water	6/2019; 9/2020	6/2019; 9/2020	6/2019; 9/2020	6/2017; 8/2017; 8/2018;
	Number of samples			
Acartia spp.	5	15	6	-
Centropages hamatus	-	20	5	3 M)
Pseudocalanus spp.	5	-	-	2 (D)
Temora longicornis	2	15	5	4 (M)
Evadne normannii	5	8	5	2 (S), 1 (M)
Stations	Å17, Släggö	BY2, BY5	BY15, BY16	BY31
Maximum depth of stations	348m, 75m	46m, 90m	241m	449m
Zooplankton collection depths	0-25 m (S)	0-30 m (S)	0-30 m (S)	0-30 (S), 30-60 (M), 60-90 m (D)
Literature reference	Serandour et al., 2023			Zamora-Terol et al., 2020

Supplementary Table 2. The average environmental parameters measured during each sampling event. The zooplankton collection depths are marked with letters (S = surface, M = medium, and D = deep).

Station	Location	Depth	Year	Month	Salinity	Temp. °C	Oxygen ml/l	Chlorophyll µg/l
A17	Marine	S	2019	June	32.5	10.7	6.6	1.1
Slaggo	Marine	S	2020	September	29.5	16.7	5.1	1.2
BY2	SBS	S	2019/2020	June/Sept.	8.1/8.3	12.5/16.4	7.4/5.8	1.3/2.2
BY5	SBS	S	2019/2020	June/Sept.	7.5/7.7	11.6/15.9	7.8/6.2	1.2/2.0
BY15	CBS	S	2019/2020	June/Sept	7.1/7.2	9.9/14.7	8.3/6.5	1.7/3.0
BY16	CBS	S	2020	September	7.2	14.7	6.5	3.0
BY31	BY31	S	2017	June/August	6.7/6.5	9.0/12.9	7.8/6.1	2.1/2.1

BY31	BY31	M	2017	June/August	7.4/7.7	4.2/4.4	7.5/5.8	0.3/0.4
BY31	BY31	D	2017	June/August	9.6/9.7	5.1/5.2	1.7/0.8	0.3/0.2

Supplementary Table 3. Number of ASVs in Syndiniales Group I, II, III, and IV associated with zooplankton hosts and water samples, and total SG ASVs for each location.

Taxa	Location	SG I	SG II	SG III	SG IV	Total SG ASVs
Acartia	Marine	3	-	-	2	5
Acartia	SBS	7	2	-	1	10
Acartia	CBS	9	2	-	-	11
Centropages	SBS	8	2	-	2	12
Centropages	CBS	6	2	-	-	8
Centropages	BY31	5	1	-	1	7
Evadne	Marine	4	3	-	-	7
Evadne	SBS	7	2	-	-	9
Evadne	CBS	8	2	-	-	10
Evadne	BY31	2	1	-	-	3
Pseudocalanus	Marine	1	-	-	2	3
Pseudocalanus	BY31	3	2	-	3	8
Temora	Marine	4	1	-	-	5
Temora	SBS	8	3	-	-	11
Temora	CBS	10	2	-	-	12
Temora	BY31	5	2	-	-	7
Water	Marine	52	88	11	2	153
Water	SBS	20	38	11	2	71
Water	CBS	18	20	8	1	47
Water	BY31	37	77	22	1	137
Total		123	203	38	5	369

Supplementary Table 4. Generalized Linear Model (GLM) output of SG I, II, III, and IV relative read abundances in the water and zooplankton host samples as a function of environmental factors. Predictor variables include salinity, temperature, chlorophyll, and dissolved oxygen concentration. The intercepts of the models represent the baseline abundance of each SG when environmental parameters are at zero. Significant *p*-values ($p < 0.05$) are in bold font type. The used link function was quasipoisson. The lower part of the table summarizes the goodness-of-fit for each GLM model using deviance reduction as a measure of explained variation. SG IV explains the most variation (58.7%), followed by SG III (43.1%). SG I captures 17.2%, while SG II has the lowest explanatory power at 10.6%.

		Estimate	Std. error	t-value	*p* -value
**SG I**	(Intercept)	-0.41	0.57	-2.48	**< 0.05**
	Salinity	0.00	0.01	0.66	0.51
	Chlorophyll	0.02	0.14	0.16	0.88
	Temperature	-0.06	0.02	-2.27	**< 0.05**
	Oxygen	0.23	0.07	3.45	**< 0.01**
**SG II**	(Intercept)	-8.93	3.42	-2.61	**< 0.05**
	Salinity	0.04	0.03	1.49	0.14
	Chlorophyll	-0.51	0.56	-0.92	0.36
	Temperature	0.12	0.10	1.23	0.22
	Oxygen	0.78	0.35	2.21	**< 0.05**
**SG III**	(Intercept)	-1.42	1.20	-1.18	0.24
	Salinity	0.00	0.05	0.02	0.99
	Chlorophyll	2.15	0.42	5.08	**< 0.01**
	Temperature	-0.51	0.10	-5.16	**< 0.01**
	Oxygen	-0.13	0.15	-0.88	0.38
**SG IV**	(Intercept)	-3.05	2.11	-1.45	0.15
	Salinity	-0.45	0.29	-1.46	0.15
	Chlorophyll	-9.13	6.11	-1.45	0.14
	Temperature	2.08	1.24	1.68	0.10
	Oxygen	-1.63	0.71	-2.28	**< 0.05**

Model	Null Deviance	Residual Deviance	Explained Deviance (%)
**SG I**	55.77	46.16	17.2%
**SG II**	39.82	35.59	10.6%
**SG III**	5.68	3.23	43.1%
**SG IV**	54.95	22.70	58.7%
