# Supplementary material for: Diet composition and environmental niche drive parasitic Syndiniales interactions with crustacean zooplankton
Source: ISME Commun. 2025 Dec 24;6(1):ycaf248. doi: 10.1093/ismeco/ycaf248 (PMC13245725; doi:10.1093/ismeco/ycaf248)
Supplement: Supplementary_Table_4_ycaf248 [file supplementary_table_4_ycaf248.docx]

**Supplementary Table 4.** Generalized Linear Model (GLM) output of SG I, II, III, and IV relative read abundances in the water and zooplankton host samples as a function of environmental factors. Predictor variables include salinity, temperature, chlorophyll, and dissolved oxygen concentration. The intercepts of the models represent the baseline abundance of each SG when environmental parameters are at zero. Significant *p*-values (*p* < 0.05) are in bold font type. The used link function was quasipoisson. The lower part of the table summarizes the goodness-of-fit for each GLM model using deviance reduction as a measure of explained variation. SG IV explains the most variation (58.7%), followed by SG III (43.1%). SG I captures 17.2%, while SG II has the lowest explanatory power at 10.6%.

|  |  | Estimate | Std. error | t-value | *p* -value |
| --- | --- | --- | --- | --- | --- |
| **SG I** | (Intercept) | -0.41 | 0.57 | -2.48 | **0.015** |
|  | Salinity | 0.00 | 0.01 | 0.66 | 0.51 |
|  | Chlorophyll | 0.02 | 0.14 | 0.16 | 0.88 |
|  | Temperature | -0.06 | 0.02 | -2.27 | **0.025** |
|  | Oxygen | 0.23 | 0.07 | 3.45 | **< 0.001** |
| **SG II** | (Intercept) | -8.93 | 3.42 | -2.61 | **0.010** |
|  | Salinity | 0.04 | 0.03 | 1.49 | 0.14 |
|  | Chlorophyll | -0.51 | 0.56 | -0.92 | 0.36 |
|  | Temperature | 0.12 | 0.10 | 1.23 | 0.22 |
|  | Oxygen | 0.78 | 0.35 | 2.21 | **0.029** |
| **SG III** | (Intercept) | -1.42 | 1.20 | -1.18 | 0.24 |
|  | Salinity | 0.00 | 0.05 | 0.02 | 0.99 |
|  | Chlorophyll | 2.15 | 0.42 | 5.08 | **< 0.001** |
|  | Temperature | -0.51 | 0.10 | -5.16 | **< 0.001** |
|  | Oxygen | -0.13 | 0.15 | -0.88 | 0.38 |
| **SG IV** | (Intercept) | -3.05 | 2.11 | -1.45 | 0.15 |
|  | Salinity | -0.45 | 0.29 | -1.46 | 0.15 |
|  | Chlorophyll | -9.13 | 6.11 | -1.45 | 0.14 |
|  | Temperature | 2.08 | 1.24 | 1.68 | 0.10 |
|  | Oxygen | -1.63 | 0.71 | -2.28 | **0.025** |

| Model | Null Deviance | Residual Deviance | Explained Deviance (%) | |
| --- | --- | --- | --- | --- |
| **SG I** | 55.77 | 46.16 | 17.2% |  |
| **SG II** | 39.82 | 35.59 | 10.6% |  |
| **SG III** | 5.68 | 3.23 | 43.1% |  |
| **SG IV** | 54.95 | 22.70 | 58.7% |  |
